# Deconstructing the MELD Score in Heart Failure: The “Cardiorenal” Fallacy

**DOI:** 10.1002/clc.70371

**Published:** 2026-06-15

**Authors:** Fatima Ali, Syed Huzaifa Khan, Syed Muhammad Rayyan

**Affiliations:** ^1^ Khyber Medical University Peshawar Pakistan; ^2^ Ayub Medical College Abbottabad Pakistan

To the Editor,

I read with great interest the article by Kozluca et al., “Diuretic Response Prediction With MELD Score in Heart Failure,” which evaluates the utility of the Model for End‐Stage Liver Disease (MELD) score derivatives in predicting diuretic response (DR) among patients with decompensated heart failure [1]. While the authors present a compelling narrative framing the MELD score as a valuable index for “cardiorenal stress,” a closer inspection of the data presented in Table 3 suggests alternative interpretations regarding the mechanisms driving these results.

First, the characterization of the MELD score as a “cardiorenal” marker in this context appears misaligned with the study's own findings. If the score were truly reflecting cardiorenal syndrome, one would expect significant deviations in renal markers. However, the data reveal no statistically significant difference between poor and good DR groups regarding serum creatinine ($*p* = 0.47$) or glomerular filtration rate (GFR) ($*p* = 0.56$) [2]. In contrast, the International Normalized Ratio (INR)—a marker highly sensitive to hepatic congestion—was significantly higher in the poor response group ($*p* = 0.02$). This discrepancy strongly suggests that the predictive power observed is driven by systemic venous congestion affecting the liver, rather than intrinsic renal dysfunction. The “renal” component of the proposed cardiorenal mechanism appears statistically silent.

Second, the study highlights a critical methodological limitation often seen with composite scores. The authors advocate for the MELD score's capability to analyze “multi‐organ function.” However, the “Original MELD” score (comprising bilirubin, INR, and creatinine) failed to reach statistical significance in predicting DR ($*p* = 0.18$). The score only became predictive when sodium was added in the “MELD‐Na” derivative ($*p* = 0.008$). When a multi‐organ composite score fails, but succeeds only after the addition of a single variable, it mathematically implies that the added variable is carrying the weight of the prediction [3]. Therefore, attributing the success to the complex evaluation of multi‐organ failure is a logical fallacy; the data suggest the “MELD” component is largely redundant.

Finally, this reliance on the sodium component raises questions about clinical utility. The data shows that serum sodium alone was significantly lower in the poor response group ($*p* = 0.03$). Hyponatremia is a well‐established, potent predictor of diuretic resistance and adverse outcomes in heart failure. Since the MELD‐Na score appears to derive its statistical significance primarily from hyponatremia—while its other components (creatinine, bilirubin) show weak or no correlation—clinicians might question the value of calculating a complex hepatorenal score [4]. It appears that simple serum sodium monitoring may offer similar prognostic information without the added complexity.

## Funding

The authors have nothing to report.

## Ethics Statement

The authors have nothing to report.

## Consent

The authors have nothing to report.

## Conflicts of Interest

The authors declare no conflicts of interest.

## Data Availability

Data sharing is not applicable to this article as no data sets were generated or analyzed during the current study.
